# Variation of Gene Expression Associated with Colonisation of an Anthropized Environment: Comparison between African and European Populations of *Drosophila simulans*

**DOI:** 10.1371/journal.pone.0079750

**Published:** 2013-11-18

**Authors:** François Wurmser, Tristan Mary-Huard, Jean-Jacques Daudin, Dominique Joly, Catherine Montchamp-Moreau

**Affiliations:** 1 Laboratoire Évolution Génomes et Spéciation, CNRS UPR9034, Gif-sur-Yvette, and Université Paris-Sud, Orsay, France; 2 INRA UMR 518 MIA, Paris, France; 3 AgroParisTech, UMR 518 MIA, Paris, France; 4 UMR de Génétique Végétale, INRA, Université Paris-sud, CNRS, Gif-sur-Yvette, France; University of Poitiers, France

## Abstract

The comparison of transcriptome profiles among populations is a powerful tool for investigating the role of gene expression change in adaptation to new environments. In this study, we use massively parallel sequencing of 3′ cDNAs obtained from large samples of adult males, to compare a population of *Drosophila simulans* from a natural reserve within its ancestral range (eastern Africa) with a derived population collected in the strongly anthropized Rhône valley (France). The goal was to scan for adaptation linked to the invasion of new environments by the species. Among 15,090 genes retained for the analysis, 794 were found to be differentially expressed between the two populations. We observed an increase in expression of reproduction-related genes in eastern Africa, and an even stronger increase in expression of Cytochrome P450, Glutathione transferase and Glucuronosyl transferase genes in the derived population. These three gene families are involved in detoxification processes, which suggests that pesticides are a major environmental pressure for the species in this area. The survey of the *Cyp6g1* upstream region revealed the insertion of a transposable element, *Juan*, in the regulatory sequence that is almost fixed in the Rhône Valley, but barely present in Mayotte. This shows that *Cyp6g1* has undergone parallel evolution in derived populations of *D. simulans* as previously shown for *D. melanogaster*. The increasing amount of data produced by comparative population genomics and transcriptomics should permit the identification of additional genes associated with functional divergence among those differentially expressed.

## Introduction

The regulation of gene expression is a major, yet still poorly understood, contributor to phenotypic plasticity and adaptation to changing environments [1], [2]. Its direct and strong influence on phenotypes makes expression a major target of natural selection [3]. Large scale technologies such as microarrays and more recently RNA-seq have allowed the development of whole transcriptome comparisons of natural populations to comprehensively identify changes in expression potentially linked to adaptation [4], [5]. A diversity of organisms has been studied in this context [6]–[12].

Large scale measures of expression can also be a phylogenic marker under a neutral hypothesis, as suggested by [13]. However, such an approach is clearly limited by the plastic nature of gene expression. Overall, studies of gene expression divergence between taxa have shown a role of two major forces: local adaptation to the environment and sexual selection [8], [12], [14], [15].

The role of the environment in shaping expression patterns has been well illustrated in several studies. Oleksiak et al. [6] showed that sympatric populations of *Fundulus heteroclitus* and *F. grandis* exhibited a similar expression pattern, strongly contrasting with an allopatric *F. heteroclitus* population living in cold water. Similarly, a study on the Atlantic salmon focused on expression changes induced by environmental conditions. The authors released domestic animals into the wild and recaptured the progeny for their study, thus examining the consequences of environmental differences. They identified changes linked to water clarity and salubrity [11]. Evans et al. [16] explored changes related to salmon physiology during migration and identified a broad-scale transcriptional regulator, significantly predictive of survival. In *D. melanogaster*, Hutter et al. [10] and Muller et al. [12] found putatively adaptive differences in gene expression comparing an African and a European population. The species originates from Africa. They conclude that the patterns observed could be explained by a mutation-selection balance model. Recently, two latitudinal clines have been described in *D. melanogaster*, with allelic frequency changes and *cis*-regulation evolution [17], [18]. Parallel evolution on different continents is a strong evidence that these observations are caused by natural selection. Using Next Generation Sequencing, Kolaczkowski et al. [19] found evidence for major changes between a tropical and a temperate population, notably in regulatory regions.

A search for potentially recent adaptation to a newly invaded environment was permitted by our choice of *Drosophila simulans*, a close relative of *D. melanogaster*, which has also spread in environmentally contrasted areas. It will be interesting to assess whether *D. simulans* is showing parallel evolution due to similar environmental differences. This generalist species originates from eastern Africa, around Kenya/Madagascar [20]–[23]. It separated from *D. melanogaster* about two to three million years ago [20], [24], [25], and from its two sister species *D. sechellia* and *D. mauritiana* about 250 000 years ago [25], [26]. The worldwide spread of *D. simulans* is thought to be more recent than that of *D. melanogaster* [21]. The recentness of the invasion first led to the idea that *D. simulans* was only slightly structured, an idea originally supported by allozyme based studies [27] as well as morphometric data [28]. This pattern contrasts with what has been shown later by studies on DNA sequence variation. Using microsatellite markers, Schfl and Schlterrer [29] showed geographic structure between southern Africa and the cradle of the species. This pattern was confirmed on nuclear loci [30]. Overall, *D. simulans* shows little population structure within its presumed ancestral range (Kenya, Tanzania, Madagascar and Mayotte), while derived populations from southern or western Africa, Europe, the Middle East, North or South America show more structure [29]–[33]. Here, we examine transcriptome variations in relation to the out of Africa migration of *D. simulans*, to search for potentially recent adaptations to the newly invaded environment.

A previous study using microarrays revealed few differences between three African populations and a French one [34]. Here we used next generation sequencing as a powerful tool to examine whole transcriptome differences between a derived European population from a temperate agricultural area in the Rhône Valley (Gotheron) and an African population from a natural reserve in Mayotte, within the ancestral Afrotropical range of the species. Mayotte’s agriculture is still highly traditional, with very small parcels, and a rare use of chemicals. We sampled large populations (100 individuals per replicate) at each location to broaden our assessment of the transcriptome on a population scale.

## Methods

### Fly Collection

**Figure 1:**
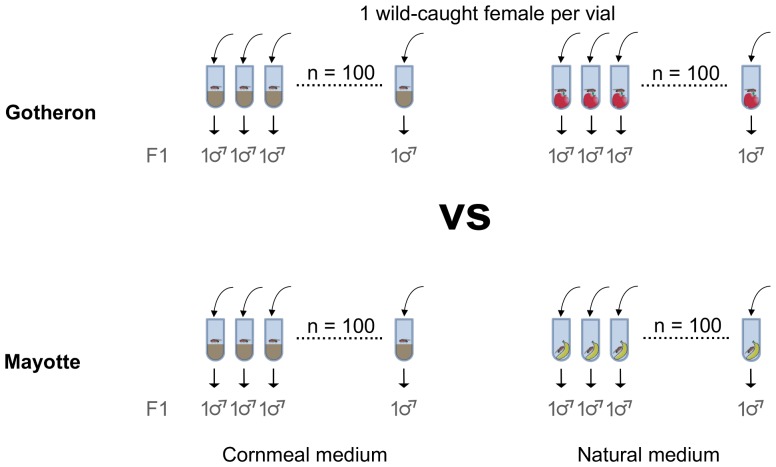
Experimental design. Each replicate involved a hundred males each from a wild-caught female. We sequenced two replicates per population, one from a cornmeal medium, and the other from a natural medium.

Adult flies were collected directly from their natural habitat, using both traps and butterfly nets. Flies from the Rhône Valley were captured in September 2009 (close to the annual population density peak of the species [35]) in an untreated apple orchard (surrounded by regularly treated parcels) located at 44°58′20″N latitude and 4°55′39″E longitude. INRA graciously provided us access to their field for collection of the French population. Flies from Mayotte were captured in November 2009 in a clearing in mid-height of the main island, located at 12°48′25″S latitude and 45°9′12″E longitude in November. In Mayotte, the collection was done in the wild and did not require specific authorisation. The species is cosmopolitan and neither endangered nor protected. For each location, we started 200 isofemale lines as follows: each wild-caught female was introduced into an individual vial containing either axenic medium (100 lines) or the local natural resource (100 lines), i.e. apple for Gotheron, banana for Mayotte. Flies were reared at 25°C. First generation offspring males (G1) were collected at emergence and placed in vials for aging during 5 days on the same medium as parents. Each vial contained a maximum of 25 flies. Males were then instantly frozen at −80°C (see Figure 1 for experimental design).

#### RNA extraction

For each location and culture medium, RNA was extracted separately from four pools of 25 G1 males, each from a different isofemale line. RNA was extracted using Nucleospin RNA II kit from Macherey-Nagel according to manufacturer’s instructions, and checked for concentration and quality using both Nanodrop (Thermo Scientific) and microchip electrophoresis (Experion, Bio-Rad). For each population and culture medium, the four RNA extracts obtained were pooled, thus providing two replicates per population for sequencing. RNA was precipitated in 100% ethanol for transport.

#### Library preparation and sequencing

Library preparation and sequencing were performed by the biotechnological company GATC Biotech (GATC inc.). From the total RNA samples, poly(A) RNA was prepared, and was then used for cDNA synthesis. cDNA was synthesized using an oligo(dT)-linker primer and M-MLV H reverse transcriptase for first strand synthesis. The reaction conditions were chosen such that the length of the first-strand cDNAs was ranged from a 100 to 500 nt. For Illumina sequencing, the cDNAs ranged from 250 to 450 bp were eluted from preparative agarose gels. Library quality was verified on the Shimadzu MultiNA microchip electrophoresis system. 3′cDNA sequencing was performed on Illumina Genome Analyzer according to manufacturer’s instructions (the technique used is thus 3′Digital Gene Expression). Fragment length was 34 bases. All data are available in the Gene Expression Omnibus database under the accession number GSE49127.

#### Mapping

The selection of 3′UTRs prior to sequencing allowed us to significantly enhance the depth of quantification. Low quality sorting and adapter trimming was done using seqclean software (options: -v -l 32 -y 7 -x 90). Mapping was performed by GATC Biotech using ELAND software supplied by Illumina, using 32 kmer and allowing up to two mismatches (6.25% error rate). We chose to map the sequences first to the *D. melanogaster* genome, and only secondly, for those of the sequences that did not map at the first step, to the *D. simulans* genome. This double mapping strategy was chosen since the genome of *D. simulans* is not as well annotated and assembled as the genome of *D. melanogaster*. Reads mapped to *D. simulans* were reassociated with their *D. melanogaster* ortholog to simplifiy the analysis (notably the Gene Ontology analysis). Flybase orthology was verified using a divergence analysis, and was checked/corrected with best reciprocal Blast [36] when necessary (divergence>21%, corresponding to 93% alignment of randomly associated genes). Short reads may result in a poor mapping for highly diverged sequences. For that to affect our results, there needs to be a strong divergence between the two populations, so that there is a differential efficiency in mapping. Short reads are fine up to 3% divergence for expression analysis [37]. Very few genes will display that level of divergence difference from the reference genome [30], and thus this bias should be minimal.

#### PCR and transposon assessment protocol

We performed a long PCR using the Phusion enzyme from Finnzymes, following manufacturer’s instructions. We also designed a triplex PCR, with two primers flanking the insertion site, and one primer inside the transposon. The primers were designed so that without insertion, the fragment would be 300 bp long, whereas in the presence of the element, the amplified fragment would be 600 bp long. We used the Gotaq enzyme from Promega. All heterozygotes along with two homozygotes of each category were verified by Sanger sequencing on an ABI 3130.

#### Statistical analysis of differential expression

Bacterial contamination of the natural medium in Mayotte led us to exclude 268 immunity related genes from the analysis, in order to focus on more relevant gene categories. These genes were selected according to their Gene Ontology association, and excluded from the analysis prior to statistical analysis.

In the recent literature, several articles advocated the use of overdispersed or extended Poisson distribution procedures for the analysis of NGS data [38]–[40]. These procedures take into account both the discrete (counting) and overdispersed nature of the data to be handled. In the present paper, we performed a 2 step analysis, under the following hypotheses:

- most of the genes are non differentially expressed (NDE),
- genes with similar mean expression levels have similar dispersion levels.

**Figure 2:**
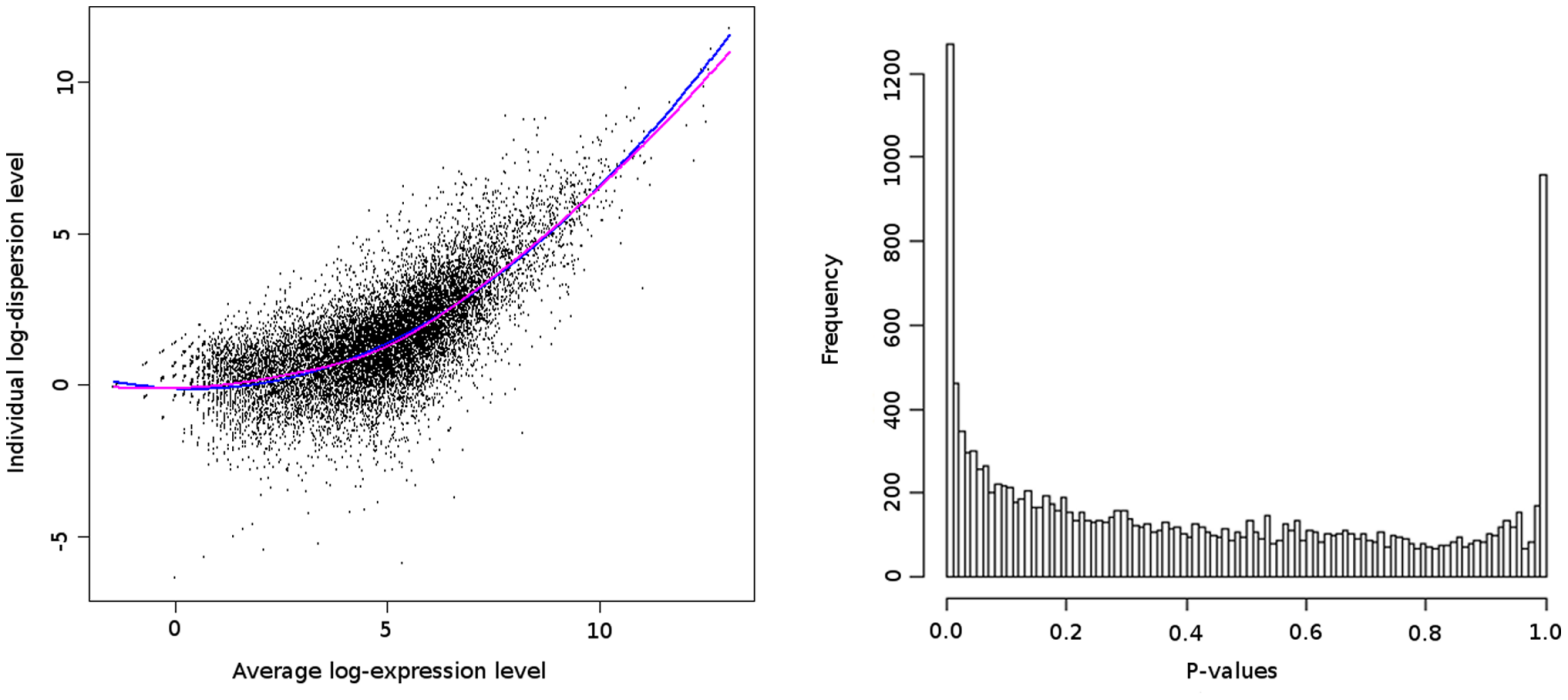
Left: Local estimation of dispersion. Dispersion parameter estimates 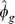 as a function of the mean expression 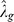 (log-scale). Each point corresponds to a gene. The purple and blue curves represent the Loess and Quadratic Regression estimates, respectively. **Right:** Histogram of the p-values for the G vs M comparison.

In the first step, a gene-by-gene analysis is performed using the following overdispersed Poisson model:
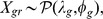
where 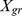 is the observed expression of gene 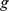 for replicate 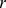, and 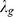 and 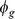 are the mean and dispersion parameters associated with gene 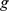, respectively. Note that in this model the mean expression level 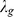 does not depend on the condition, which is relevant for most genes under hypothesis (i). All four replicates may then be used to obtain an estimate 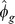 of the dispersion parameter 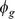. For NDE genes the variance is unbiasedly estimated, while it is over-estimated for DE genes. Under hypothesis (ii), a more robust estimation 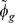 of 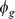 can be obtained using a Loess local estimation of 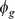 on genes with similar average expression levels. Figure 2 (left) displays the dispersion parameter as a function of the mean expression of the genes, along with the Loess curve of estimates 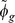 (in purple). The Loess is very close to the quadratic curve (in blue) that corresponds to the quadratic relationship between mean and variance of the overdispersed Poisson that is usually assumed in many alternative procedures [39], [41].

In the second step, a gene-by-gene analysis is performed using another overdispersed Poisson model:
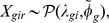
where 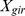 is the observed expression level of gene 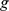 in condition 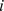 for replicate 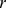, 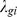 is the mean expression of gene 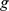 in condition 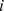, and 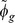 is the dispersion parameter estimated in the previous step. Likelihood Ratio Tests (LRT) can then be performed to test the population effect “Gotheron vs Mayotte” using the following contrast:



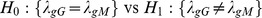
and obtain p-values. Figure 2 (right) displays the p-value distribution corresponding to these tests. The excess of values on the right of the histogram is due to the first step of the analysis that leads to an slight overestimation of the dispersion parameter (since most but not all genes are non DE). Importantly, as mentioned in [41], this overestimation decreases the power of the procedure, but does not affect the control of Type I error. Once the p-values are obtained, a classical BH correction [42] is performed to control the FDR (FDR = 0.05).

#### Consistency filtering of differentially expressed genes

Because a particular food medium may have a strong interaction with a small subset of genes, we applied a consistency filter. For genes overexpressed in Gotheron, we checked whether both replicate were consistently greater in Gotheron than in Mayotte. We did the same for genes overexpressed in Mayotte. 50 genes were filtered out, while 794 genes remained after filtration.

#### Statistical analysis of gene clustering

In order to test whether differentially expressed genes were clustered on the genome, we estimated the distribution of the average closest distance between 844 random genes from 10,000 Monte Carlo runs, assuming no clustering. We then compared the observed mean distance to this distribution and estimated the probability of observing an equal or shorter mean distance under the null hypothesis 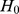: no clustering of differentially expressed genes.

#### Gene ontology

We examined lists of differentially expressed genes using FuncAssociate [43], an online tool looking for under/over-representation of ontology terms.

## Results and Discussion

We compared the transcriptome profile of an Afrotropical and a temperate European population of *D. simulans*. We used two replicates, each made up of a hundred first generation males from a hundred different females collected in the wild (Figure 1). We observed 495 genes overexpressed in Gotheron compared with Mayotte and 349 overexpressed in Mayotte compared with Gotheron. The differences lay essentially in detoxification genes.

### Mapping Reads to *Drosophila* Genomes

Sequencing produced about 30 million reads per replicate. About 15% of the reads were eliminated by quality filters. Of the remaining reads, about 65% were successfully mapped on the *D. melanogaster* genome and 20% were then mapped on the *D. simulans* genome. About 15% of the cleaned reads remained unmapped. We could then use about 20 million mapped reads per replicate. We assessed expression for 15,090 genes, 12,716 with a *D. melanogaster* ID, and 2,374 with a *D. simulans* ID (with no *D. melanogaster* ortholog annotated and no significant result using reciprocal blast). According to Flybase release notes [44], the genome of *D. simulans* is composed of around 15,000 to 17,000 genes. We therefore have a good coverage of the genome, although it is likely that some genes are still described by two IDs, despite our efforts to get rid of this possible bias. However, we feel this problem remains marginal and affects mainly poorly annotated genes for which we will not be able to analyse the function anyway.

### Population Differentiation: a Mix of Drift and Local Adaptation?

Our analysis revealed 794 differentially expressed genes between the Gotheron population and the Mayotte population (Table S1). 469 genes are overexpressed in France, while 325 are overexpressed in Mayotte. This difference may be due to the stronger positive environmental selection to which the French population is exposed. Differentially expressed genes between populations may essentially reflect two phenomena: local adaptation, or stochastic differentiation between populations. Analysis of the molecular functions of genes helps differentiate between the two processes. We analysed the genes differentially expressed using Gene Ontology tools (FuncAssociate, [43]) to reveal overrepresented attributes.

#### Overexpression in mayotte: reproduction related genes

**Figure 3:**
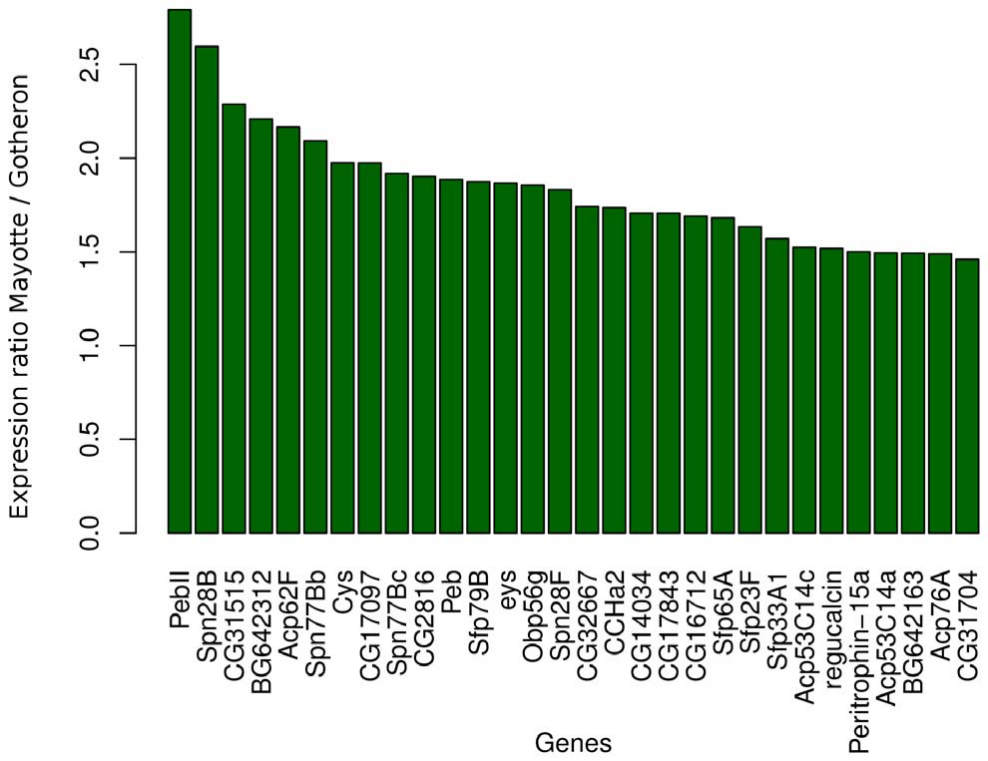
Fold ratio of differentially expressed reproduction-related genes. Barplot of the ratio of expression of Mayotte over Gotheron for reproduction related genes overexpressed in Mayotte.

Among the 325 genes underexpressed in Gotheron compared with Mayotte, overrepresented Gene Ontology terms (Table 1) point to changes in reproductive process. Within the 30 differentially expressed genes involved in the process (some of these had only *D. simulans* IDs after automatic processing and therefore were not analysed via the gene ontology tool, but added manually because they shared the same annotated function), we mainly find genes encoding seminal fluid proteins (Figure 3), described as protease inhibitors. Many proteolysis regulators have been described in both male and female reproductive tracts in Drosophila, [45], which suggests they play an important role in male-female co-evolution. Their role is to prevent degradation of seminal fluid proteins by the female [46]–[48]. In fact, proteolysis modulates the function of male proteins in female post-mating response such as ovulation, sperm storage, egg-laying and sperm usage [49]. Protease may be a general regulatory switch used by males to quickly activate many female responses after mating. In *D. melanogaster*, males who produce and transfer larger quantities of seminal fluid proteins have a significantly higher reproductive success in a competitive environment [50].

**Table 1:** Gene ontology terms for genes overexpressed in Gotheron compared with France.

N	X	P-value	GO ID	GO term
15	75	3.11E-10	GO:0032504	multicellular organism reproduction
15	77	4.60E-10	GO:0000003	reproduction
18	145	1.63E-8	GO:0005615	extracellular space
18	250	4.60E-5	GO:0044421	extracellular region part

With N the number of genes with the term in the query; X the number of genes with the term in the genome; P-value of the significance of the overrepresentation of the term in query compared to genome, processed with FuncAssociate [43]; GO ID and GO term, respectively the identifier and the corresponding term of Gene Ontology.

The environment in Mayotte may be more reproductively competitive than in Gotheron, because it is less variable throughout the year, allowing persistence at higher density of the local drosophila population. Indeed, average monthly temperatures in Mayotte range from 24°C to 28°C while in the Rhône Valley, monthly temperatures range from 5°C to 23°C.

The overexpression of protease inhibitor genes revealed here in *D. simulans* males is consistent with differentiation of expression in the populations via sexual selection. Sexual selection creates strong positive selection for co-evolution of reproductive functions in populations and species of *Drosophila* [51], a phenomenon also described in other insects and mammals [45].

### Overexpression of Genes in Gotheron: Detoxification of Xenobiotics

Among the 469 genes overexpressed in Gotheron, fifteen gene ontology terms were significantly overrepresented (Table 2). A detailed analysis of the terms showed that three sets of genes, representing three gene families are described by the ontology terms. The first family is the Cytochrome P450 gene family. The second is the glutathione transferase (GST) gene family. The third is UDP-glucosyltransferases (UGT). All three families are involved in xenobiotic detoxification [52]–[56]. *Cyp6g1* is actually the only gene overexpressed in Europe in our study as well as in males and females of *D. melanogaster* [10], [12]. Why is there only one common gene? There might be several reasons. First, it is possible that *D. melanogaster* and *D. simulans* have adapted differently to their environment, and that their process of invasion did not involve the same set of genes (at least in terms of expression differentiation). The second hypothesis is that our European population from the Rhône Valley was more exposed to pesticides than the *D. melanogaster* populations collected from Leiden (Netherlands). This hypothesis does not seem likely, as both locations are surrounded by agricultural areas. However, the treatments could be very different in the two areas, since the Rhône Valley is mainly composed of fruit and vegetable fields, while Leiden’s agriculture is mainly flower plantations.

**Table 2:** Gene ontology terms for genes overexpressed in Gotheron compared to Mayotte.

N	X	P-value	GO ID	GO term
29	164	5.91E-13	GO:0009055	electron carrier activity
14	38	1.78E-11	GO:0004364	glutathione transferase activity
23	140	7.03E-10	GO:0020037	heme binding
18	85	7.45E-10	GO:0005792	microsome
18	85	7.45E-10	GO:0042598	vesicular fraction
23	141	8.12E-10	GO:0046906	tetrapyrrole binding
25	178	3.74E-09	GO:0016705	oxidoreductase activity, acting on paired donors, […]
54	666	9.25E-09	GO:0016491	oxidoreductase activity
26	201	1.06E-08	GO:0005506	iron ion binding
18	101	1.36E-08	GO:0005624	membrane fraction
18	104	2.20E-08	GO:0005626	insoluble fraction
14	62	2.46E-08	GO:0016765	transferase activity, transferring alkyl or aryl […] groups
18	107	3.49E-08	GO:0000267	cell fraction
10	34	1.72E-07	GO:0015020	glucuronosyltransferase activity
42	533	9.81E-07	GO:0055114	oxidation-reduction process

With N the number of genes with the term in the query; X the number of genes with the term in the genome; P-value of the significance of the overrepresentation of the term in query compared to genome, processed with FuncAssociate Berriz2003; GO ID and GO term, respectively the identifier and the corresponding term of Gene Ontology.

#### Chromosomal location of differentially expressed genes

We assessed clustering of differentially expressed genes using a Monte Carlo approach. Clustering of differentially expressed genes was non significant (p-value = 0.81 with 10,000 iterations). However, Cytochrome P450, GSTs and UGTs are highly clustered gene families [52], [54], [55]. This co-location raises the question of co-regulation of clustered genes. For example, five differentially expressed genes are located around the 10,760,000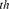 base of chromosome 2R (*Cyp6a17*, *Cyp6a23*, *Cyp6a20*, *Cyp6a21*, *Cyp6a8*). Within this cluster, are four other cytochrome genes, none of which is differentially expressed. Three hypotheses can explain this pattern. First, there is indeed co-regulation, but these genes also have their own regulation that counteracts the global regulation of the cluster. Second, these genes are co-localised merely for historical reasons (i.e. tandem gene duplication), but do not share common regulation. This hypothesis is favoured by the literature [57], although it does not exclude the first hypothesis. Third, there is coordinated change of expression within a cluster, but the power of our test could not detect differentiation of other genes of the cluster. The latter hypothesis can be ruled out: we checked expression for other genes in the cluster and our data clearly indicates that their expression is similar in both populations.

#### Glutathione transferase enzymes, an adaptation to local environment

**Figure 4:**
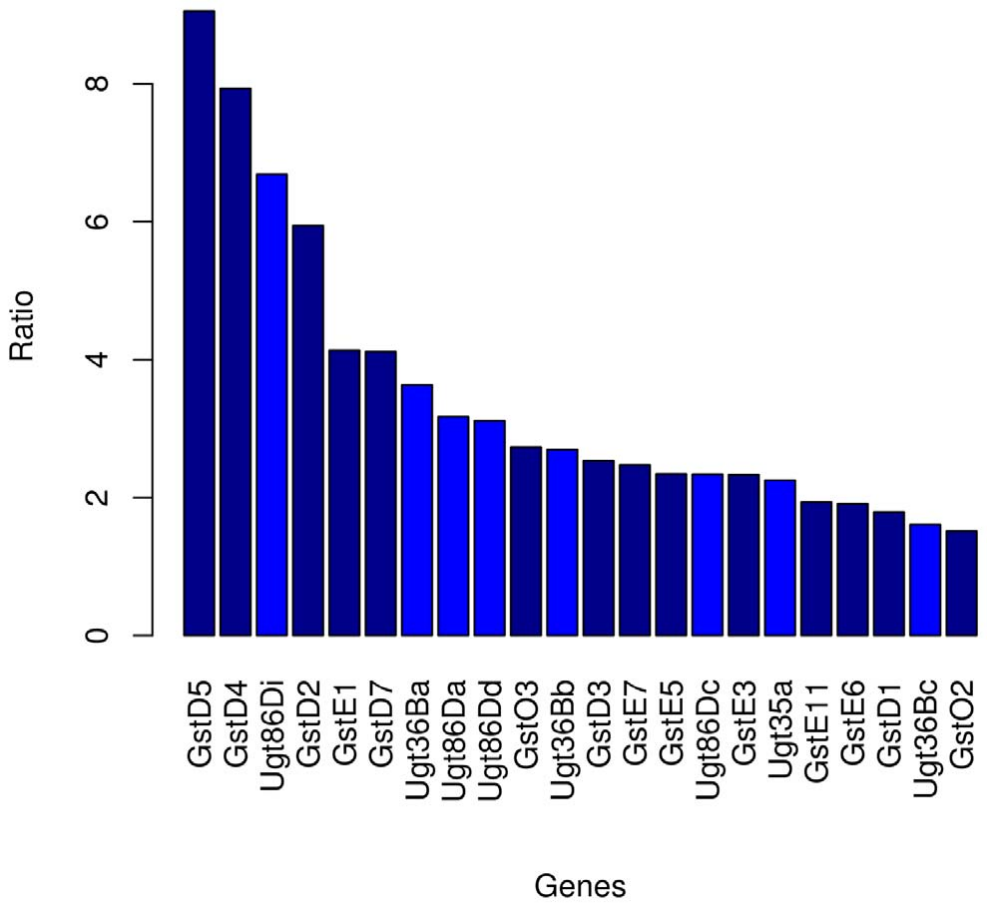
Fold ratio of differentially expressed GST/UGT genes. Barplot of the ratio of expression of Gotheron over Mayotte for Glutathione and glucuronosyl transferase genes overexpressed in the Rhône Valley.

The French population shows stronger expression than the Mayotte population for six Delta, six Epsilon and two Omega GSTs (Figure 4). In *D. melanogaster*, GST genes belong to a large family composed of 38 members [55]. These genes are assigned to different classes according to sequence homology and immunological reactions [53], [58]. Two of these classes, namely Delta and Epsilon GSTs, are insect specific and have undergone a major expansion via gene duplication. *D. melanogaster* has nine Delta and fourteen Epsilon functional GSTs [55]. Indeed, these two subfamilies have expanded their number independently in *D. melanogaster* and *Anopheles gambiae*. This suggests that these enzymes play a major role in the species adaptation to their environment. The multiplication of the gene copies should have expanded the range of targets GSTs are able to detoxify [59]. Our observation is consistent with the idea that adaptation within insect species also occurs via regulatory changes [7], [12], [60]. These genes are thus good candidates for expression adaptation due to the presence of xenobiotics (pesticides) in the environment.

#### Glucosyltransferase enzymes, detoxification of xenobiotics

In Gotheron, eight UGT genes were overexpressed. *D. melanogaster* presents about 33 UGT enzymes in its genome [54]. These enzymes also have a role in detoxification, although not well functionally characterised. As for GSTs, UGTs represent a likely adaptation to an anthropised environment. Their role in detoxification has been shown in the mosquito *Aedes aegypty* using artificial selection for pesticide resistance [61].

#### The Cytochrome P450 gene family suggests selection for pesticide resistance

**Figure 5:**
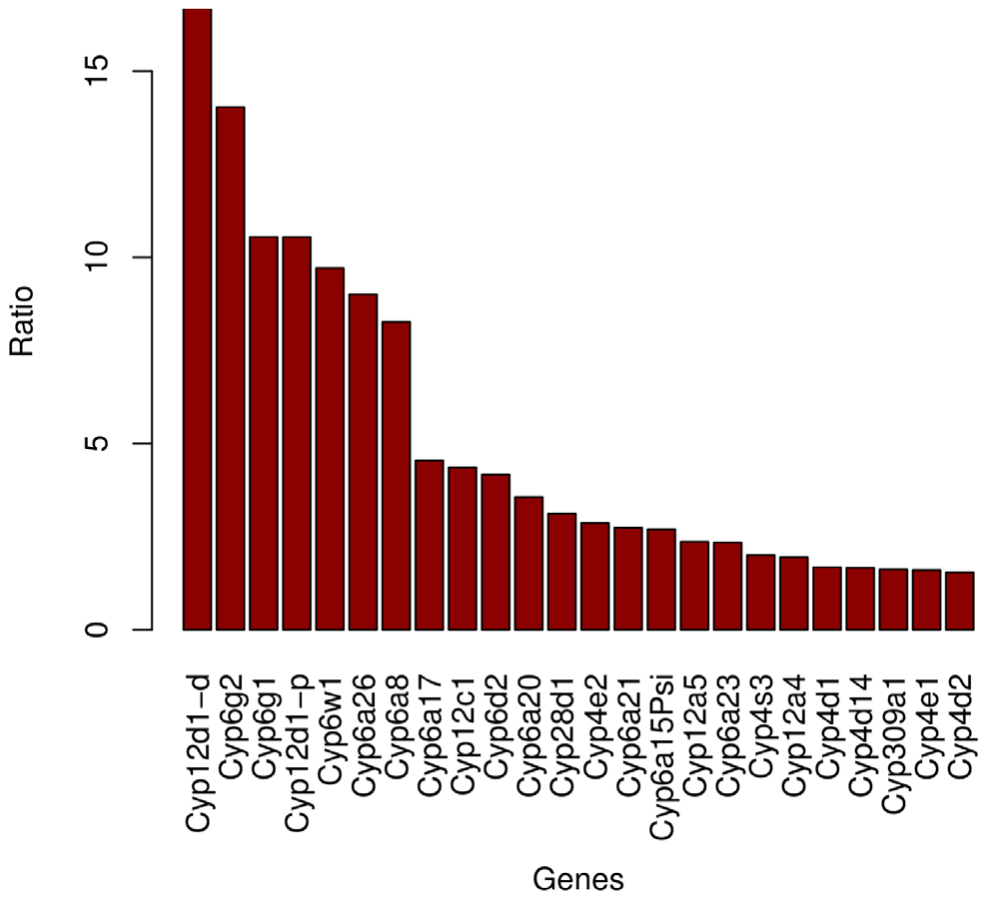
Fold ratio of differentially expressed CYP450 genes. Barplot of the ratio of expression of Gotheron over Mayotte for Cytochrome P450 genes overexpressed in the Rhône Valley.

Among the genes overexpressed in the population from the Rhône Valley compared with the population from Mayotte, 24 genes are Cytochrome P450 (Figure 5). Three additional genes have Cytochrome related functions (*CG2065*, *CG1319*, *CG18522*). P450 are among the genes with the highest fold ratio (7 out of the 10 leading genes), and all of them are key components of the detoxification machinery [44]. This family is composed of approximately 85 functional genes in *D. simulans* [52], [62]. It is a very pleiotropic gene family, with roles ranging from detoxification of xenobiotics to hormone regulation. For example, many detoxification genes of this family are underexpressed in the specialist species *Drosophila sechellia* compared with *D. simulans* [34], [63]. Specialisation reduces the diversity of toxins to which the species is exposed. Constraints on this gene family are thus relaxed, allowing a breakdown of expression [34], [63], as well as a large number of pseudogenisation [62], [64]. Although the drosophila we collected in the Rhône valley came from an orchard where pesticides are not used, the area is surrounded by fields where they are spread on a regular basis. It is likely that those genes have undergone genetic changes in regulation due to environmental constraints.

#### Cyp6g1: a major player in the detoxification process

One gene consistently overexpressed (about ten times more) in the Gotheron population compared with the Mayotte population was *Cyp6g1*, a cytochrome gene located on the chromosome arm 2R. *Cyp6g1* is an emblematic gene. It has been thoroughly studied in *D. melanogaster*, where increased expression is strongly linked to broad pesticide resistance, including resistance to DDT [65], [66] which is considered a good marker of a general role in insecticide resistance [67]. The 10 fold ratio of expression between our *D. simulans* populations, argues for a major role in pesticide resistance in this species too.

We analysed the regulatory region of *Cyp6g1*, looking for a transposon insertion, as previously observed in an upregulated allele found in a Californian population [68]. A long PCR with primers flanking the regulatory region revealed an insertion polymorphism. Sequencing the beginning of the insert in several individuals from the French population, we identified a *Juan* insertion located 10 base pairs away from the insertion site of the *Doc* element described by Schlenke and Begun [68]. We then assessed by PCR the frequency of the *Juan* insertion in our two populations. In France, out of 47 G1 males (each from a different wild caught female), 43 were homozygous for the insertion, while four were heterozygotes. In Mayotte, 45 males showed no insertion, and two were heterozygotes. The frequency of the insertion is therefore estimated between 90% to 99% (95% confidence interval) in the Rhône Valley and between 0.3% to 7% (95% confidence interval) in Mayotte. The population from California is nearly fixed (98% frequency) for the *Doc* element insertion, an insertion correlated with an increase in *Cyp6g1* expression as well as a relative resistance to DDT (evidence is still controversial) [68]. The low prevalence of the insertion in Mayotte suggests a cost of the insertion for pesticide-free populations, as indeed has been discussed in *D. melanogaster* [67]. However, this hypothesis is quite controversial [69]. An alternative explanation is that the derived allele arrived only recently in the ancestral range. Alternatively, there may be an ongoing selection for this allele due to evolution of human habits in Mayotte. Further population genetics studies should address this issue to document what appears to be a case of parallel evolution in *D. melanogaster* and *D. simulans*.

Indeed a detailed analysis of the locus in *D. melanogaster* described the progressive appearance of new alleles by gene duplication and transposon insertion. Each new allele leads to a better fitness in the presence of pesticides [67] and an increase in expression [69], [70].

In *D. melanogaster*, derived alleles are present in North Africa and close to fixation in American, European and Asian populations, but rare or even absent from eastern/southern Africa [66], [67].

Such an example of parallel evolution due to a major role in resistance is not unique: the *Resistance to dieldrin* locus, which harbours a mutant linked to insecticide resistance was shown to have arisen independently in different insect species, and even multiple times in *Tribolium casteneum* [71], [72]. This raises questions about the variety of ways to achieve a new phenotype.

## Conclusion

We compared large samples of a population from the ancestral range with a population from an invaded area of *D. simulans*, using a transcriptome-wide approach. We identified gene families linked with local adaptation via expression modifications. The major response observed involves detoxification genes, of the Cytochrome P450, Glutathione transferase and Glucosyltransferase gene families. Pesticide exposure seems to be the major selective force under which expression has evolved between these populations, as observed with the example of *Cyp6g1*. However, numerous genes could not be linked to an obvious functional divergence between populations. As ever increasing numbers of comparative analyses of genome and transcriptome variation will be conducted on natural populations of drosophila and other insect species, it should become possible to assess whether these genes evolved under neutral evolution or natural selection during the invasion of new environments.

## Supporting Information

Table S1
**List of differentially expressed genes.** Columns in order: Flybase Identifier, Expression levels for the four replicates, p-value, FDR corrected p-value, mean expression level, dispersion index 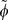.(XLS)
